# Residual Gas Adsorption and Desorption in the Field Emission of Titanium-Coated Carbon Nanotubes

**DOI:** 10.3390/ma12182937

**Published:** 2019-09-11

**Authors:** Huzhong Zhang, Detian Li, Peter Wurz, Yongjun Cheng, Yongjun Wang, Chengxiang Wang, Jian Sun, Gang Li, Rico Georgio Fausch

**Affiliations:** 1 Science and Technology on Vacuum Technology and Physics Laboratory, Lanzhou Institute of Physics, Lanzhou 730000, China; janehuge@126.com (H.Z.); chyj750418@163.com (Y.C.); wyjlxlz@163.com (Y.W.); zhz252@126.com (J.S.); ligangcasc510@163.com (G.L.); 2 Physics Institute, University of Bern, 3012 Bern, Switzerland; peter.wurz@space.unibe.ch (P.W.); rico.fausch@space.unibe.ch (R.G.F.)

**Keywords:** carbon nanotubes, residual gas adsorption, residual gas desorption, field emission

## Abstract

Titanium (Ti)-coated multiwall carbon nanotubes (CNTs) emitters based on the magnetron sputtering process are demonstrated, and the influences of modification to CNTs on the residual gas adsorption, gas desorption, and their field emission characteristic are discussed. Experimental results show that Ti nanoparticles are easily adsorbed on the surface of CNTs due to the “defects” produced by Ar^+^ irradiation pretreatment. X-ray photoelectron spectroscopy (XPS) characterization showed that Ti nanoparticles contribute to the adsorption of ambient molecules by changing the chemical bonding between C, Ti, and O. Field emission of CNTs coated with Ti nanoparticles agree well with the Fowler–Nordheim theory. The deviation of emission current under constant voltage is 6.3% and 8.6% for Ti-CNTs and pristine CNTs, respectively. The mass spectrometry analysis illustrated that Ti-coated CNTs have a better adsorption capacity at room temperature, as well as a lower outgassing effect than pristine CNTs after degassing in the process of field emission.

## 1. Introduction

Carbon nanotubes (CNTs) have attracted great interest based on their potential industrial applications since their discovery by Iijima [1]. Among a number of proposed applications [2,3,4,5,6], CNTs exhibit important applications in field emission (FE) devices used in vacuum physics, e.g., for ion sources and vacuum gauges. Such CNT field emission devices are most promising industrially, and their practical use is an imminent prospect [7,8,9,10,11,12,13]. FE characteristics of nanotubes from a single multi-walled nanotube (MWNT) and MWNT film were first reported by Rinzler et al. and de Heer et al., respectively [14,15]. Subsequently, many CNTs FE emitters were reported to be applied in power microwave sources, ion source, electric propulsion, X-ray tubes, vacuum gauges, and more [16,17,18,19].

Generally, CNTs are very sensitive to different types of molecules in their gaseous environment, which is a promising characteristic for sensors. For instance, Pt and Au coatings on the CNTs improved the sensitivity of chemiresistors to indicate the abundance variations of H_2_, NH_3_, NO_2_, etc. [20,21]. However, in virtue of the special gas-sensitivity of CNTs, the adsorption and desorption of residual gas from CNTs could disturb the background vacuum of FE devices, change the work function of CNTs, and affect FE stability and repeatability [22,23,24,25,26]. Due to the change in residual gas coverage on the surface of field emitters, the change in work function plays an important role in electron emission, noise spectrum, and stability [27]. Sergeev et al. also formulated an adsorption–desorption model based on the Kolmogorov equation for field emitters [28]. Dean et al. heated a single-walled nanotube (SWNT) to high temperature to clean the surface, which led to a lower emission current but a better low-frequency stability [23]. Cho et al. had reported that CNTs modified by metallic nanoparticles contributed to extending the FE lifetime of CNTs [29]. Thus, keeping the nanotubes free from residual gases should be an effective way to improve the FE stability and reduce the outgassing of CNTs emitters in ultrahigh vacuum (UHV) electric devices. 

In this work, we report on multi-walled carbon nanotube (MWNT) films for possible application in UHV ionization gauges (IG) or mass spectrometers (MS). These CNT films are grown in a thermal chemical vapor deposition (CVD) system onto stainless-steel substrates that were pretreated by the oxidation–reduction method. The as-grown CNTs were then coated by Ti nanoparticles through the magnetron sputtering process. The interaction of CNTs with the residual gas in UHV and FE characteristics of the emitters are experimentally studied and discussed.

## 2. Materials and Methods 

The MWNTs applied in the experiment were prepared on the stainless-steel substrate via an oxidation–reduction treatment [30]. As this is different from existing chemical methods [31], we then used the magnetron sputtering technique for in situ coating of the as-grown pristine CNTs with the Ti nanoparticles. Ar^+^ plasma with 1.38 keV energy was applied to bombard the walls of MWNTs to introduce some “defects” on the surface that facilitate the adherence of Ti nanoparticles. In the experiments, the samples were divided into two groups, hereafter referred to as “Ti-CNTs #1” and “Ti-CNTs #2,” which were pretreated by Ar^+^ plasma for 40 s and 60 s, respectively. Two groups of pretreated MWNTs were then coated inside a vacuum chamber on a sample plate placed 30 cm from a rotating Ti target (4 rpm). The operation parameters of the apparatus were set as follows: microwave power of 200 W, deposition duration of 42 s, argon atmosphere of 0.3 Pa. 

Ti-CNTs #1, Ti-CNTs #2 and original pristine CNTs were all characterized by a FEI Tecnai G2 F20 high-resolution transmission electron microscope (HRTEM) (FEI Technologies Inc., Hillsboro, OR, USA) and Peikin-Elmer PHI-5702 multi-functional X-ray photoelectron spectroscopy (XPS) (Physical Electronics Inc., Chanhassen, MN, USA). A diode configuration was established using the CNT films as a cathode against a stainless-steel anode, to evaluate the FE performance of samples. An Inficon 422 quadrupole mass spectrometer (INFICON GmbH, Köln, Germany) was applied to measure the residual gas variation in the UHV chamber.

## 3. Results and Discussion

### 3.1. Characteristics of the CNT Samples

**Figure 1:**
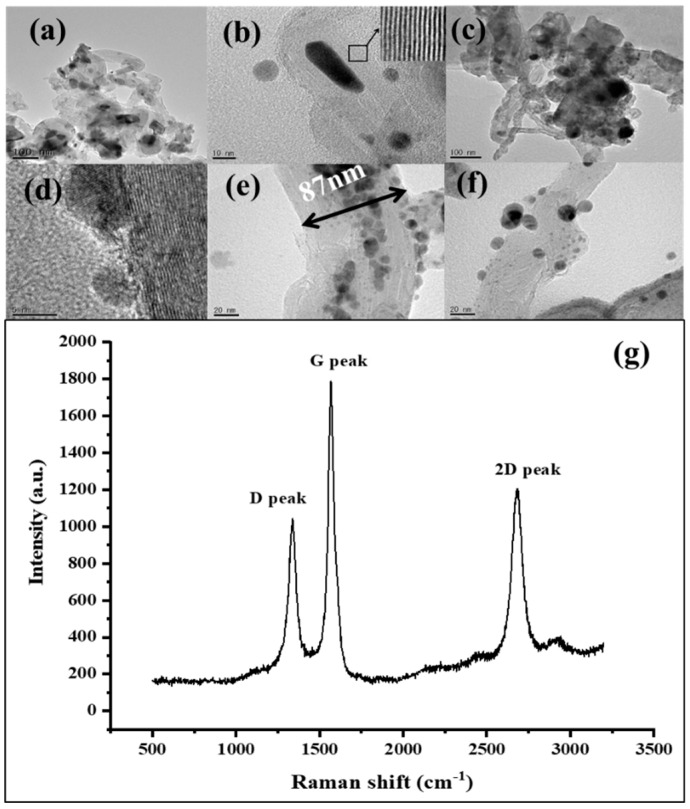
High-resolution transmission electron microscopy (HRTEM) images of (**a**) pristine MWNT, (**b**) magnification of single tube of pristine CNTs (inset: magnification of HRTEM image of outer shells.), (**c**) Ar^+^-irradiated MWNTs, (**d**) Ti nanoparticles on the normal outer wall of CNTs, (**e**) Ti nanoparticles on the larger-diameter defective CNTs, (**f**) Ti nanoparticles on the “junction” of defective CNTs, (**g**) Raman spectrum of pristine CNTs.

TEM micrographs of pristine CNTs without plasma treatment and Ti coating are presented in Figure 1a,b. CNTs with an average diameter around 40–60 nm having hollow and tubular structures are observed in Figure 1a. Most CNTs appeared to be curved or twisted together, and few amorphous carbon or catalyst nanoparticles are observed on the surface of CNTs. The pristine CNTs have a large and wide diameter distribution and good crystallinity, which can be inferred from the Raman spectrum (Figure 1g). The intensity ratio of the G peak at 1568.8 cm^−1^ and D peaks at 1338.7 cm^−1^, *I*_G_/*I*_D_, for the pristine CNT, is about 1.7, which is comparable to reported high-quality CNTs on stainless-steel alloy substrates [32,33]. The crystalline graphite shell of the pristine CNT consisted of visible lattice fringes parallel to each other (Figure 1b). Figure 1c shows HRTEM images of the Ar^+^ plasma-treated MWNTs, which should cause “defects” with more amorphous carbon and tube expansion [34]. The specific defective structures are shown in Figure 1e,f. Except for the adsorption on the normal CNTs outer wall, as shown in Figure 1d, the Ti nanoparticles with a size of 4–6 nm tended to be deposited on sites close to the “defects.”

**Figure 2:**
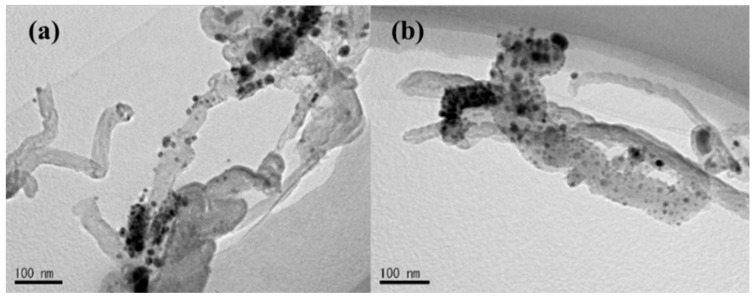
HRTEM of titanium-coated CNTs under the process of magnetron sputtering. (**a**) Ti–CNTs #1 with 40 s irradiation and 42 s DC sputtering. (**b**) Ti-CNTs #2 with 60 s irradiation and 42 s DC sputtering.

The aggregates of nanoclusters dispersed nonuniformly onto the surface of CNTs, and depend mainly on the Ti nanoparticles’ cohesion energy, Ti-CNTs’ interface energy, and the diffusion barrier value [35,36]. It is believed that “defects” produced by plasma irradiation create some vacancies that serve as nucleation sites for the adsorption of foreign molecules [37]. The distribution of Ti nanoparticles could be noticed clearly in the two Ti-CNTs samples shown in Figure 2. The Ti nanoparticles are attached onto the surface of tube sidewalls and on the tops randomly, as can be seen in Figure 2a. By extending the Ar^+^ sputter time to 60 s for Ti-CNT #2 samples, the coverage of Ti nanoparticles increased, and the Ti particles distributed randomly onto the nanotubes as shown in Figure 2b. 

**Figure 3:**
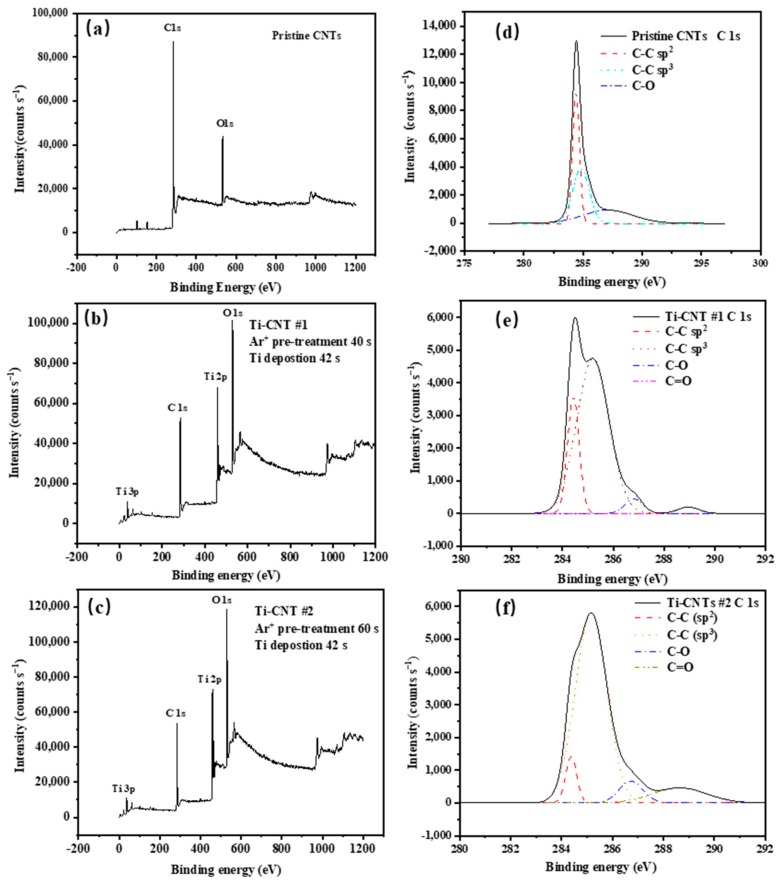
The XPS spectra of different CNT samples: (**a**) pristine CNTs, (**b**) Ti-CNTs #1, (**c**) Ti-CNTs #2, (**d**) C 1*s* states of Pristine CNTs, (**e**) C 1*s* states of Ti-CNTs #1, (**f**) C 1*s* states of Ti-CNTs #2.

The chemical bonds states of CNTs samples have been analyzed. The XPS survey scans of pristine CNTs, Ti-CNTs#1, and Ti-CNTs #2 are shown in Figure 3a–c, which clearly indicate that Ti was successfully deposited on the CNT surface. Carbon bonds were also changed in the processes of Ar^+^ irradiation and Ti coating. Figure 3d–f shows the XPS spectra of C 1*s* states for three samples, in which several deconvoluted peaks, including C–C sp2, C–C sp3, C–O, and C=O bonds, were assigned to the binding energy of 284.4 eV, 285.1 eV, 286.5 eV, and 288 eV, respectively. For the pristine CNTs, the C1*s* spectrum reveals a high concentration of C–C sp2 hybridized bonds because of the highly crystalline qualities of CNTs. The broad asymmetric tail towards higher binding energy is consistent with C–C sp3 hybridization and C–O bonds. Additionally, the results of Figure 3e,f show that the C–C sp2 hybridized bonds decreased, while C–C sp3 hybridized bonds increased after the Ti deposition. It can be deduced that Ar^+^ ions irradiation produced the amorphous “defects” shown in Figure 1. Ti-CNTs #2 undergoing 60 s Ar^+^ irradiation with higher C–C hybridized sp3 intensity have more defects on their structure.

**Figure 4:**
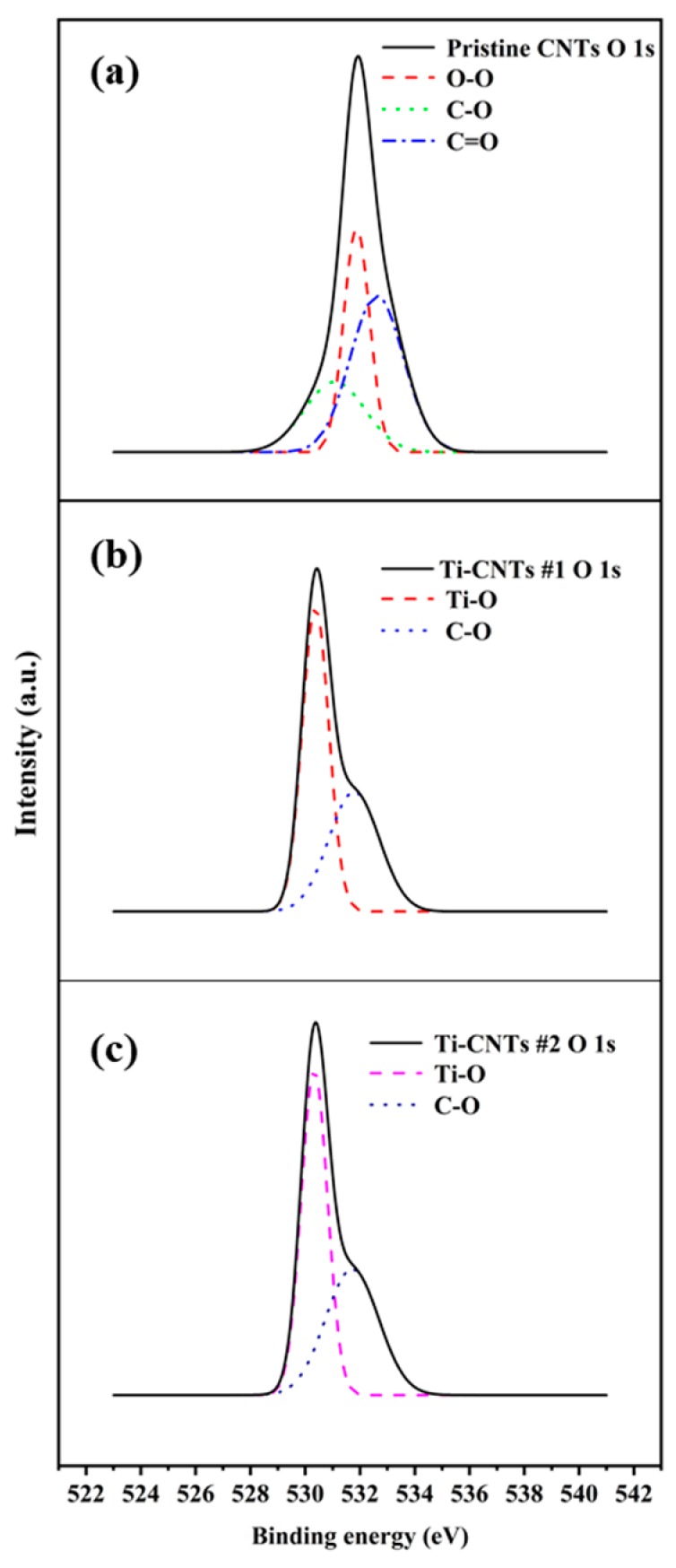
The XPS spectra of O 1*s* states of (**a**) pristine CNTs, (**b**) Ti-CNT #1, and (**c**) Ti-CNT #2.

The structural “defects” of CNTs provided the adsorption sites for Ti nanoparticles that modify the chemical bonding structure. Because of the oxidation tendency of Ti nanoparticles, the chemical binding among Ti, C, and O changed. The alteration of O 1*s* states is shown in Figure 4. For the pristine CNTs, O–O at a binding energy of 532 eV (originating from an adsorbed O_2_ molecule) is observed. Moreover, some O atoms formed C–O bonds and C=O bonds at a binding energy of 531.5 eV and 532.5 eV, respectively, whereas, for Ti-CNTs #1 and Ti-CNTs #2, particular chemical shifts of the main peak are noted. This could be caused by Ti–O binding from the titanium oxide at a binding energy of 530.2 eV. Meanwhile, Ti–O binding could prevent the formation of C–O bonds and C=O bonds, which are the key factors that affect the FE stability and repeatability of CNTs [29].

**Figure 5:**
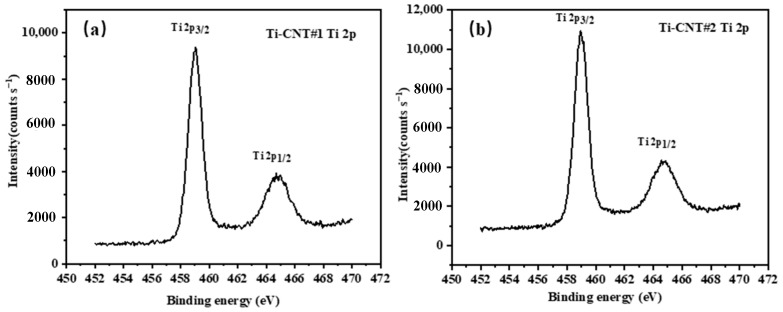
The XPS spectrum of Ti 1*p* states of (**a**) Ti-CNT #1 and (**b**) Ti-CNT #2.

Figure 5 shows the Ti 2*p* states of Ti-CNT #1 and Ti-CNT#2. Both Ti-CNTs had the peaks of Ti bonds corresponding to Ti 2*p*3/2 and Ti 2*p*1/2 spin-orbital doublets at 458.5 eV and 464.8 eV, respectively. The sharp and symmetrical peaks of Ti 2*p* states were close to those of bulk TiO_2_, indicating the presence of Ti clusters [38]. Combining these with the Ti–O bonds at the binding energy of 530.2 eV in Figure 4, it can be deduced that Ti was significantly oxidized. The Ti-CNTs’ surface layers, composed of Ti–O bonds, should protect the CNTs’ structure from being damaged by molecules of the ambient gas, i.e., O_2_ and H_2_O, which could be confirmed from the chemical shifts in Figure 4. 

**Figure 6:**
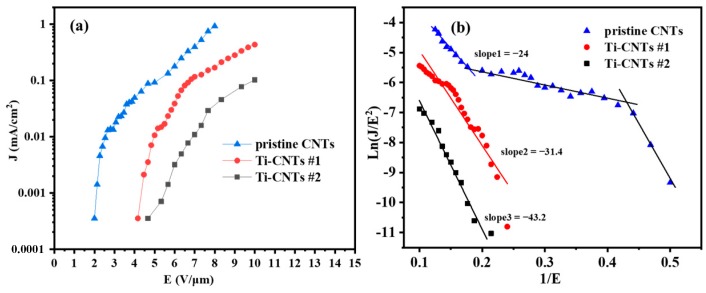
Field emission performance of different emitters. (**a**) J–E electron emission properties, and (**b**) F–N plots of these data.

Using diode devices, pristine CNTs, Ti-CNT #1, and Ti-CNT #2 were fabricated and tested in UHV. The CNTs samples of 28.3 mm^2^ emission area were grounded with a ballast resistor of 24 kΩ connected in series. It was kept 150 μm away from the anode. To ensure reliable FE operations, all the samples were put in a vacuum at ~10^−6^ Pa to be conditioned in advance at a FE current of ~30 μA for 1 h. Figure 6 shows the FE current density–electric field (*J*–*E*) curves and the corresponding Fowler–Nordheim (F–N) plots. The FE of pristine CNTs shows a lower turn-on field (2.6 V/μm) and higher emission current than that of Ti-coated CNTs (5.0 V/μm for Ti-CNT #1 and 6.9 V/μm for Ti-CNT #2) in Figure 6a. The higher turn-on field could be explained by the loss of chemisorbed surface states and accompanying resonant-enhanced tunneling current due to the adsorption of residual gas by Ti nanoparticles rather than CNTs [22]. Moreover, the presence of H dipoles is well known to reduce the work function of CNTs, but the high adsorption capacity for H of Ti decreases the number of H dipoles on the surface of the CNTs [39,40]. Meanwhile, due to partial oxidization, the Ti–O bond was recognized to increase the work function of Ti-coated CNTs. It was theoretically estimated to increase by ~0.6 eV [29]. In addition, plasma treatment may also result in a radius increase of CNT tips, which weakens the field enhancement effect. The tube expansion shown in Figure 1c, in agreement with earlier work [34], could result in a smaller field enhancement factor. Both the increase of work function and the decrease of field enhancement factor lead to the slope change of fitted F–N curves in Figure 6b.

In Figure 6b, the pristine CNTs indicate typical FE characteristics, with three emission stages of FE, including adsorption-dominated emission at low current range, intermediate range, and intrinsic emission at high current range [23,41,42]. Ti-coated CNTs have better agreement with the F–N theory than pristine CNTs, as shown by the “orthodoxy test” proposed by Forbes [43,44]. Assuming that all the samples have intrinsic emission, for estimated work function 4.8 eV and calculated slopes (Figure 6b), the scaled barrier field *f* ranges are (0.15–0.62), (0.25–0.59), and (0.20–0.43) for pristine CNTs, Ti-CNTs #1, and Ti-CNTs #2, respectively. For the orthodox emission, the values *f* are supposed to range in 0.15–0.44. Thus, we conclude that the discrete Ti compound layer adsorbed most of the residual gas and kept the surface of nanotubes clean, which contributed to such orthodox FE without adsorption dominated emission for Ti-CNTs #2. However, the adsorbates modified the work function to cause “unorthodox” emission for Ti-CNT #1 and pristine CNTs.

**Figure 7:**
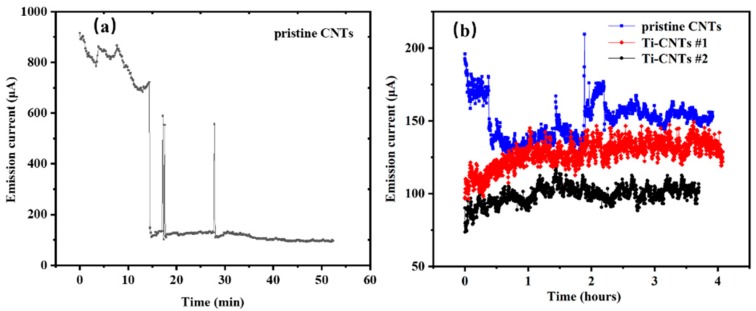
Emission current summary of Ti-coated CNTs and pristine CNTs over time. (**a**) Aging conditioning of pristine CNTs during a short period. (**b**) Stability comparison.

To make a more definitive comparison of three CNT samples, the stability tests were conducted in the 10^−7^ Pa UHV chamber and the emission current was recorded under constant voltage mode. As shown in Figure 7a, a significant drop in current occurred in the first 15 min of conditioning, resulting from the direct interaction of residual gas with CNTs and their structural degradation. Along with the desorption of residual gas, the emission current gradually became stable. This did not happen to the Ti-coated CNTs in 4 h of stability tests. As shown in Figure 7b, even after the initial conditioning, there are still discontinuous jumps in the emission current for pristine CNTs, which could lead to the electric disturbance or failure of devices. Neglecting the significant discontinuous jumps, the average emission current of pristine CNTs is 150.6 μA (anode voltage: 1644 V) with a relative standard deviation (RSD) of 8.6%. In contrast, Ti-coated CNTs showed better stability. The emission current is kept to 127.5 μA (anode voltage 1830 V) and 99.2 μA (anode voltage: 2500 V), with a smaller RSD of 7.2% and 6.3% for Ti-CNT #1 and Ti-CNT #2, respectively. More importantly, it could be seen that the emission current of Ti-coated CNTs did not show any abrupt jumps. The emission current increases slightly and becomes stable gradually. The stability improvement of Ti-coated CNTs is mainly caused by the desorption of residual gas, as well as the Ar^+^ plasma treatment [27,45]. Pristine CNTs with adsorbed residual gas influence chemisorbed surface states and increase the 1/f noise component. Employing the flicker noise interpretation described by Schottky [46], the 1/f noise spectrum of the gaseous adsorbate can be described by the adsorption–desorption model [47]. The flicker noise was proved to be dependent on the residual gas coverage, where the 1/f part is enlarged owing to the shot noise [27]. 

### 3.2. Gas Adsorption of Different CNTs at Room Temperature

**Figure 8:**
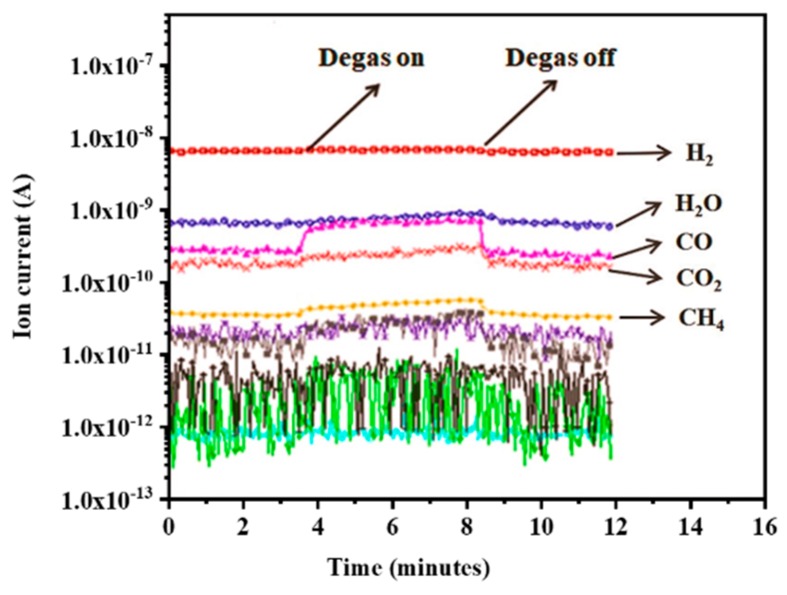
Residual gas composition of UHV chamber background after 24 h degassing, corresponding to a total pressure of 3 × 10^−6^ Pa.

To further analyze the influence of Ti-coated CNTs on the ultrahigh vacuum (UHV), pristine CNTs, Ti-CNTs #1, and Ti-CNTs #2 were successively installed in the UHV chamber via feedthrough. The composition of background residual gases was first measured while the vacuum chamber was degassed at 260 °C for 24 h, as well as the IG (extractor gauge, Leybold Corp., Cologne, Germany) degassed several times. Figure 8 shows the typical temporal development of residual gas species in the UHV chamber of 3 × 10^−6^ Pa. It is also well known that CO, CO_2_, and CH_4_ are the typical residual gas components or chemical reaction products desorbed from the IG and vacuum chamber walls, dominated by H_2_ and H_2_O. While the partial pressure in the chamber is stable, the IG, using an Ir–Y_2_O_3_ filament, was degassed via electron bombardment at an emission current of ~80 mA. As shown in Figure 8, electron-stimulated desorption (ESD) of CO^+^ increases dramatically during degassing of the filament, but other gases, including CO_2_^+^, CH_4_^+^, and H_2_^+^, rise only slightly [48]. 

**Figure 9:**
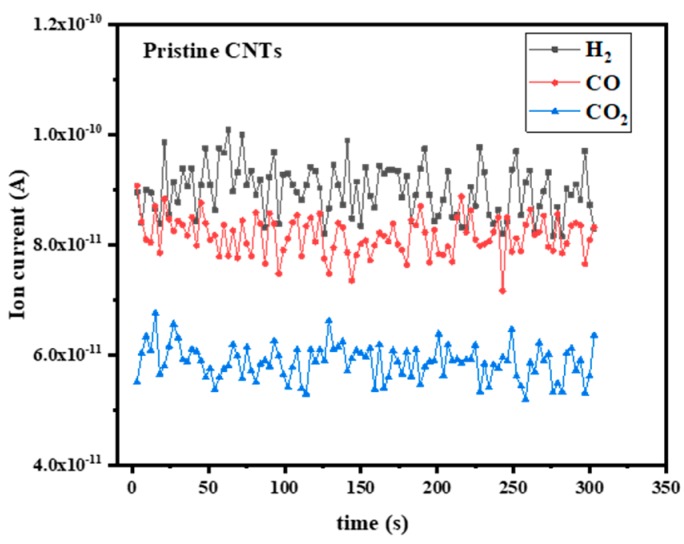
Background residual gas in UHV at room temperature for pristine CNTs without FE operation, corresponding to a total pressure of 1 × 10^−6^ Pa.

The pristine CNTs emitter was placed into the vacuum chamber without FE operation at room temperature. Figure 9 shows the gas adsorption capability of pristine CNTs in the vacuum chamber. The vacuum chamber was not degassed at high temperature but pumped over a long period of time. Although the H_2_O was the main component, the background partial pressure of the residual gases H_2_, CO, and CO_2_ decreased apparently. The weak van der Waals interaction between pristine CNTs and residual gases played a key role in the results. 

**Figure 10:**
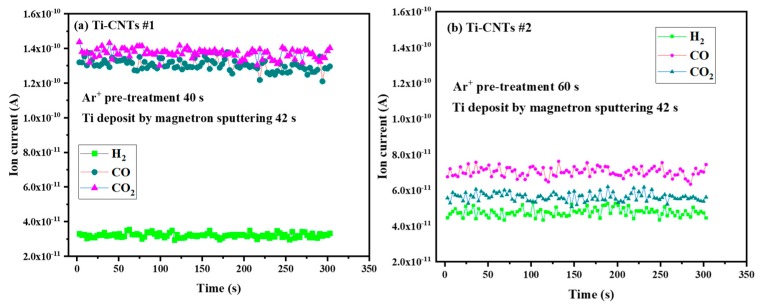
Background residual gas of titanium coated CNTs in UHV of 1 × 10^−6^ Pa at room temperature and without FE operation. (**a**) Ti-CNTs #1, (**b**) Ti-CNTs #2.

For the Ti-CNTs #1 and Ti-CNTs #2, Ti atoms (a transition metal) were prone to react with H_2_ and oxocarbons. Ti nanoparticles slightly changed the atomic level structure of CNTs in combination with residual gases. In the case of no electron emission, the adsorption performance on Ti-CNTs is shown in Figure 10. Both H_2_^+^ signals decreased to the level of 10^−11^ A at room temperature, while Ti-CNTs #1 and Ti-CNTs #2 were placed in a vacuum chamber. In particular, owing to more Ti coverage, Ti-CNT #2 showed a larger adsorption capacity than Ti-CNTs #1 for residual gases. The CO^+^ and CO_2_^+^ signals were also distinctly reduced for Ti-CNT #2. This means that the residual gas was mainly adsorbed by Ti compound to keep CNTs away from the gas adsorbates. The results could also explain the good intrinsic FE characteristic of Ti-CNT #2. 

### 3.3. Gas Desorption of CNTs under Field Emission

**Figure 11:**
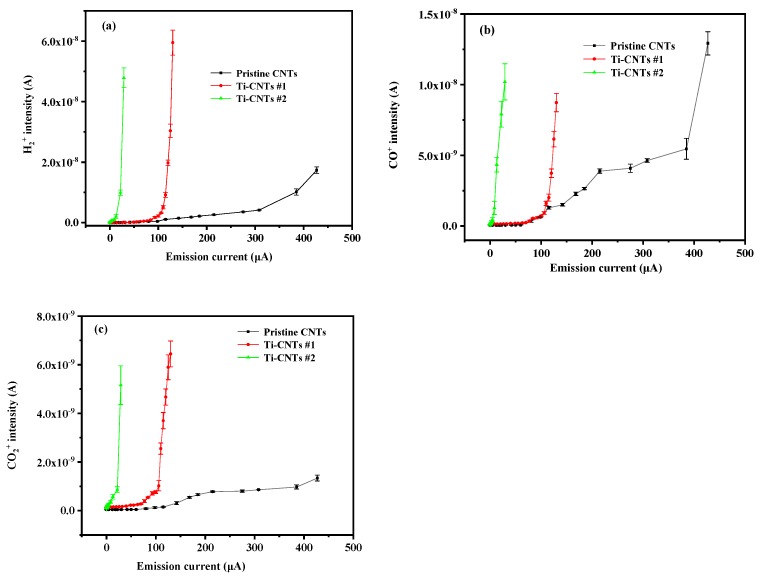
Gas desorption along with variation of FE current for pristine CNTs, CNTs #1 and CNTs #2. (**a**) H_2_^+^ intensity, (**b**) CO^+^ intensity, (**c**) CO_2_^+^ intensity.

The gas desorption was tested in the processes of FE for pristine CNTs, Ti-CNT #1, and Ti-CNT #2. Variations in the main reactive gases in UHV, i.e., H_2_, CO, and CO_2_, were detected, as shown in Figure 11. In Figure 11a, the H_2_^+^ signal of pristine CNTs, with a background level of 5.97 × 10^−11^ A, increases to 1.74 × 10^−8^ A, along with the emission current rising from 0 μA to 427 μA. It is indicated that the H_2_ adsorbed on the surface of pristine CNTs was released slowly along with the increase in the FE current, which may influence the FE performance and UHV. In contrast, H_2_^+^ in the Ti-CNTs #1 and Ti-CNTs #2 were released rapidly under a low current. The ion signal of H_2_^+^ increases from 3.22 × 10^−11^ A to 5.95 × 10^−8^ A when the emission current varies from 0 μA to 123 μA for Ti-CNTs #1. For Ti-CNTs #2, when the FE current increases from 0 μA to 30 μA, H_2_ dissociates faster than the two above-mentioned samples. 

In Figure 11b, CO does not show a significant increase for the pristine CNTs until the emission current is higher than ~60 μA, and therefore most of the released CO should be typical ESD ions from the stainless-steel anode [48]. By contrast, CO instantly increased to the same level as pristine CNTs for Ti-modified samples, originating from ESD and gas dissociation effects. Moreover, as shown in Figure 11c, CO_2_ increases slightly for pristine CNTs due to the low background pressure of CO_2_ in UHV. However, CO_2_ is prone to combine with Ti and Ti oxides, so the CO_2_ on Ti-CNT #1 and Ti-CNT#2 was also dissociated from the surface of Ti nanoparticles more than pristine CNTs. 

A knowledge of the adsorption–desorption properties of Ti-CNTs would be helpful to reduce the outgassing of CNTs emitters in the UHV electronic devices. The Ti-CNTs #2 and pristine CNTs were placed into a UHV chamber to conduct degassing experiments. The pressure variations, along with the emission current, were recorded in Table 1. When the emission current was set to 500 μA, Joule heating of the emitters caused degassing of the CNTs and made the pressure rise from 6.98 × 10^−7^ Pa to 7.73 × 10^−7^ Pa for Ti-CNTs #2 and from 4.16 × 10^−7^ Pa to 4.51 × 10^−7^ Pa for pristine CNTs. Then the current was stabilized to 500 μA for 10 min of degassing. The pressure for Ti-coated CNTs decreased from 7.73 × 10^−7^ Pa to 6.96 × 10^−7^ Pa; in contrast, for pristine CNTs, it increased from 4.51 × 10^−7^ Pa to 4.55 × 10^−7^ Pa. Then the emission current of both emitters was adjusted to a typical current (200 μA) for IG and MS. There was a significant difference between the two emitters 10 min later. The final working pressure variation Δ*p* (*p*_2_′ − *p*_u_) is 6 × 10^−9^ Pa and −2 × 10^−8^ Pa for the pristine CNTs and Ti-CNTs #2, respectively. The results support our assertion that the low outgassing property of Ti-CNTs will limit the background disturbance and allow for accurate measurements in a UHV environment [49].

## 4. Conclusions

We investigated the influence of Ti coating of MWNTs on the adsorption of residual gas in a UHV environment and its later release during operation. Ti-coated CNTs utilizing magnetron sputtering technology enhanced the adsorption capacity of residual gas based on the chemical activity of Ti nanoparticles. The partial pressure of H_2_, CO, and CO_2_ was reduced significantly by Ti-CNTs at room temperature. The process could prevent the contamination of the CNTs surface, which would lead to good FE consistency with the F–N theory and excellent FE stability as well. Ti-CNTs prevent the electric noise or failure of devices caused by discontinuous jumps in the current emission. Moreover, degassing by Joule heating in the process of FE allows Ti nanoparticles to release adsorbed gases rapidly, which provides a way to effectively degas the CNTs emitter. It is thus a promising field emitter and should find applications in UHV measurement devices. 

Ti coating of CNTs has been shown to have a potential use in practical applications involving CNTs as a FE electron source. The stability of Ti-CNTs emitters over thousands of hours and their outgassing properties in IG and QMS should be studied in further research work.

## Figures and Tables

**Table 1:** Pressure variation during the degassing of Ti-CNTs #2 and pristine CNTs.

Pressure (time)	Ti-CNT#2 (Pa)	Pristine CNTs (Pa)	Emission Current (µA)
*p*_u_ (*t*_0_)	6.98 × 10^−7^	4.16 × 10^−7^	0
*p*_1_ (*t*_1_)	7.73 × 10^−7^	4.51 × 10^−7^	500
*p*_1_*′* (*t*_1_ + 10 min)	6.96 × 10^−7^	4.55 × 10^−7^	500
*p*_2_ (*t*_2_)	6.92 × 10^−7^	4.27 × 10^−7^	200
*p*_2_*′* (*t*_2_ + 10 min)	6.78 × 10^−7^	4.22 × 10^−7^	200
Δ*p* (*p*_2_′ − *p*_u_)	−2.00 × 10^−8^	6.00 × 10^−9^	200
